# An Exceptionally Facile Two-Step Structural Isomerization and Detoxication via a Water-Assisted Double Lossen Rearrangement

**DOI:** 10.1038/srep39207

**Published:** 2016-12-23

**Authors:** Feng Li, Chun-Hua Huang, Lin-Na Xie, Na Qu, Jie Shao, Bo Shao, Ben-Zhan Zhu

**Affiliations:** 1 State Key Laboratory of Environmental Chemistry and Eco-toxicology, Research Centre for Eco-environmental Sciences, the Chinese Academy of Sciences, Beijing 100085, PR China; 2 Linus Pauling Institute, Oregon State University, Corvallis, OR 97331, USA

## Abstract

*N*-hydroxyphthalimide (NHPI), which is best known as an organocatalyst for efficient C-H activation, has been found to be oxidized by quinoid compounds to its corresponding catalytically active nitroxide-radical. Here, we found that NHPI can be isomerized into isatoic anhydride by an unusually facile two-step method using tetrachloro-1,4-benzoquinone (TCBQ, *p*-chloranil), accompanied by a two-step hydrolytic dechlorination of highly toxic TCBQ into the much less toxic dihydroxylation product, 2,5-dichloro-3,6-dihydroxy-1,4-benzoquinone (chloranilic acid). Interestingly, through the complementary application of oxygen-18 isotope-labeling, HPLC combined with electrospray ionization quadrupole time-of-flight and high resolution Fourier transform ion cyclotron resonance mass spectrometric studies, we determined that water was the source and origin of oxygen for isatoic anhydride. Based on these data, we proposed that nucleophilic attack with a subsequent water-assisted Lossen rearrangement coupled with rapid intramolecular addition and cyclization in two consecutive steps was responsible for this unusual structural isomerization of NHPI and concurrent hydroxylation/detoxication of TCBQ. This is the first report of an exceptionally facile double-isomerization of NHPI via an unprecedented water-assisted double-Lossen rearrangement under normal physiological conditions. Our findings may have broad implications for future research on hydroxamic acids and polyhalogenated quinoid carcinogens, two important classes of compounds of major chemical and biological interest.

Halogenated quinones are a group of toxicological intermediates that can cause various deleterious effects *in vivo*^1^,^2^. More than a dozen halogenated quinones, which are suspected bladder carcinogens, were recently identified as chlorinated disinfection byproducts in both drinking and swimming pool water^3^. Tetrachloro-1,4-benzoquinone (TCBQ) is one of the major genotoxic and carcinogenic quinoid metabolites of the widely used wood preservative pentachlorophenol (PCP)^4^. PCP has been detected in at least one fifth National Priorities List sites identified by the US EPA and is classified as a group 2B environmental carcinogen by the IARC (International Association for Research on Cancer)^5^,^6^. TCBQ has also been shown as a reactive oxidation intermediate or product in processes employed to oxidize or remediate PCP in various enzymatic and chemical systems^4^,^5^,^6^,^7^,^8^. TCBQ itself has been widely applied as a fungicide as well as a dehydrating or oxidizing agent (often called *p*-chloranil).

Considerable interest in hydroxamic acids has been generated recently due to their ability to inactivate various enzymes, such as lipoxygenase and metalloprotease, causing transition metal-mediated oxidative damage. Some hydroxamates (such as deferoxamine (*1*) and suberoylanilide hydroxamic acid (*2*)), have been used to clinically treat iron-overload diseases and cancer, respectively^9^,^10^,^11^.


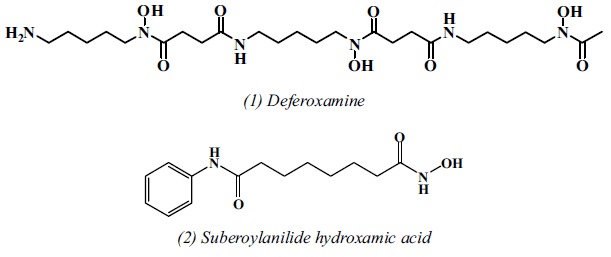


*N*-hydroxyphthalimide (NHPI) is a very unique hydroxamic acid, with two carbonyl groups linked to the nitrogen atom. NHPI is known to be used with certain co-catalysts to generate phthalimide *N*-oxyl radical (PINO), the key active organo-catalytic species for efficient C-H activation and subsequent oxygenation of hydrocarbons with dioxygen^12^,^13^,^14^,^15^. Therefore, we expected that a similar pathway may apply to the reaction between NHPI and TCBQ to produce the radical intermediate PINO in our system. According to our previous study^16^, we proposed an alternative pathway in which NHPI may attack TCBQ via nucleophilic substitution to initially form a transient intermediate NHPI-NO-TrCBQ (here, we use NHPI-NOH to refer to NHPI), followed by homolysis of the N-O bond, forming N- and O-centered radicals. However, to our surprise, neither the redox nor the nucleophilic substitution/homolysis pathway was observed during the reaction between NHPI and TCBQ. Through complementary applications of oxygen-18 isotope-labeling, high-performance liquid chromatography combined with electrospray ionization quadrupole time-of-flight mass spectrometry (HPLC-ESI-Q-TOF-MS) and high resolution Fourier transform ion cyclotron resonance mass spectrometry (FT-ICR-MS) studies, we found that TCBQ induced an unusually facile two-step isomerization of NHPI to isatoic anhydride (IA) via a water-assisted double Lossen-type rearrangement coupled with rapid intramolecular nucleophilic addition under normal physiological conditions.

## Results and Discussion

**Figure 1:**
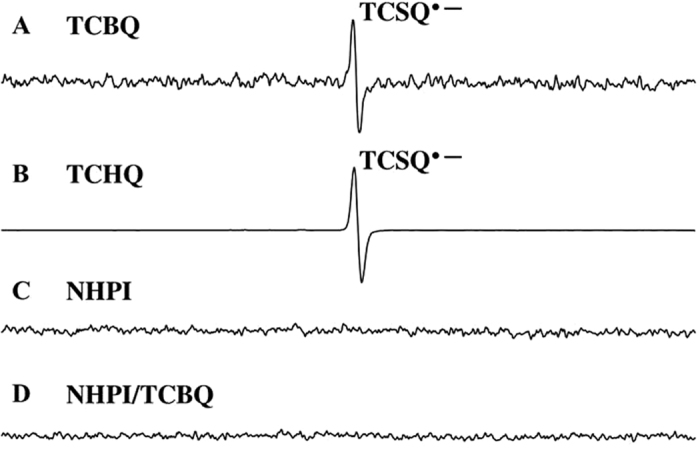
ESR spectra by incubating 100 mM DMPO with TCBQ and NHPI. (**A**) TCBQ, 0.2 mM. (**B**) NHPI, 0.4 mM. (**C**) NHPI, 0.4 mM, TCBQ, 0.2 mM. The ESR spectra were recorded 1 min after the addition of the chemicals at room temperature under normal lighting conditions. All reaction mixtures were air-saturated for the ESR experiments. The central signal in the spectrum for TCBQ was identified as the TCSQ^·−^ with a g value of 2.0056.

As mentioned above, we first attempted to determine whether TCBQ oxidizes NHPI to generate its corresponding nitroxide radical PINO under normal physiological conditions (room temperature, pH 7.4 phosphate buffer), as reported previously^15^, or if it reacts with NHPI to produce N- and O-centered radicals via nucleophilic substitution coupled by homolytic decomposition^16^. However, no new radicals (*N*- and *O*-centered radicals) were observed during the reaction between TCBQ and NHPI, as measured by the direct ESR method or secondary ESR spin-trapping using DMPO as the trapping agent (Fig. 1). The central ESR signal was identified as the tetrachlorosemiquinone anion radical (TCSQ^·−^) for TCBQ alone. Adding NHPI to TCBQ decreased the signal of this radical (Fig. 1). These results suggest that the reaction between NHPI and TCBQ was neither a redox reaction nor a nucleophilic substitution coupled with homolysis as we originally expected. Instead, we found that the reaction between NHPI and TCBQ led to a remarkable enhancement of TCBQ hydrolysis.

### NHPI markedly enhanced TCBQ hydrolysis

**Figure 2:**
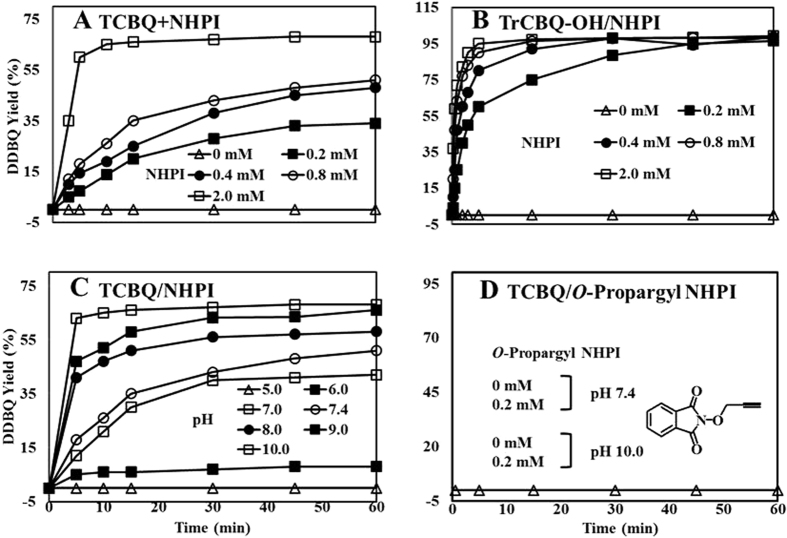
DDBQ yields of the reactions of NHPI (derivative) and TCBQ/TrCBQ-OH under different conditions. (**A**) NHPI enhanced TCBQ hydrolysis in a concentration-dependent manner. (**B**) NHPI enhanced TrCBQ-OH hydrolysis in a concentration-dependent manner. (**C**) NHPI enhanced TCBQ hydrolysis in a pH-dependent manner (TCBQ, 0.2 mM; NHPI, 0.8 mM). (**D**) *O*-propargyl NHPI did not enhance TCBQ hydrolysis. All incubation mixtures contained the indicated concentration of NHPI in phosphate buffer (100 mM, pH 7.4) at 37 °C. The reactions were initiated by the addition of TCBQ or TrCBQ-OH (0.2 mM), followed by rapid mixing. DDBQ formation was monitored using the HPLC method.

Using the HPLC method, we found that TCBQ was first spontaneously hydrolyzed to the initial transient mono-hydroxylation intermediate trichloro-hydroxy-1, 4-benzoquinone (TrCBQ-OH) and then to the final dihydroxylation product 2,5-dichloro-3,6-dihydroxy-1,4-benzoquinone (DDBQ), which is much less reactive and less toxic than TCBQ^17^,^18^ (Fig. 2A,B). We found that the rate of TCBQ hydrolysis was markedly accelerated by NHPI compared to that of its spontaneous hydrolysis. The formation of TrCBQ-OH and DDBQ, as measured by HPLC coupled with a UV-visible detector, was found to be dependent on the molar ratios between NHPI and TCBQ: at low molar ratios (≤1), TCBQ was converted primarily to TrCBQ-OH; at higher molar ratios (>1), TCBQ was first converted to TrCBQ-OH and then further to DDBQ. To test whether NHPI could also directly accelerate the slow hydrolysis of TrCBQ-OH to DDBQ, TrCBQ-OH was synthesized according to methods reported previously^19^. We found that this was indeed the case (Fig. 2B). These results suggest that the reaction proceeds sequentially, first to the initial transient intermediate TrCBQ-OH, then further to the final product DDBQ.

The rate of hydrolysis also depended on the molar ratios of NHPI/TCBQ and NHPI/TrCBQ-OH (Fig. 2A,B). The rate of hydrolysis increased as the molar ratio increased. The half time of TrCBQ-OH (0.2 mM) hydrolysis to DDBQ in the presence of 0.2, 0.4, 0.8 and 2.0 mM NHPI was found to be 3.0, 1.3, 0.6 and 0.22 min, respectively (pH 7.4). It has been shown that the half time for the spontaneous hydrolysis of TCBQ to TrCBQ-OH was 1 h, while that of TrCBQ-OH to DDBQ was 21 days^10b^. Based on these findings, our results demonstrate that NHPI enhanced the hydrolysis of TCBQ to DDBQ up to 137,000-fold (TCBQ, 0.2 mM; NHPI, 2.0 mM).

### The free N-hydroxy phthalimide anion was essential for the enhancement of TCBQ hydrolysis

Further investigation revealed that the rate of NHPI-mediated hydrolysis of TCBQ to DDBQ also depends on the pH of the buffer. TCBQ was not hydrolyzed to DDBQ at pH ≤ 5, however, as the pH increased, the rate of hydrolysis progressively increased (Fig. 2C). These results indicate that the reactive form of NHPI (pK_a_ = 7) is likely the free *N*-hydroxy phthalimide anion. To test this hypothesis further, the generation of the free anionic oxygen was blocked using *O*-propargylated NHPI. As expected, *O*-propargyl NHPI abolished the acceleration of TCBQ hydrolysis (Fig. 2D). These results clearly demonstrate that the free *N*-hydroxy phthalimide anion is essential for the enhancement of TCBQ hydrolysis.

### The major reaction product of NHPI was identified as isatoic anhydride, a structural isomer of NHPI

**Figure 3:**
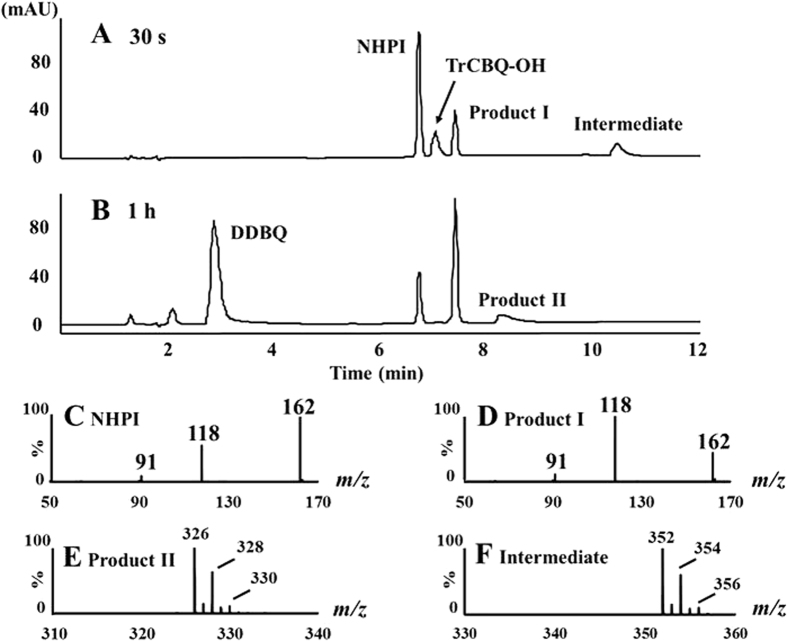
Analysis of the NHPI/TCBQ reaction products of using the HPLC-MS method. (**A,B**) The HPLC profile of the reaction of TCBQ (0.2 mM) with NHPI (0.4 mM) in phosphate buffer (pH 7.4, 0.1 M). (**C**) The ESI-Q-TOF-MS-MS spectrum of NHPI. (**D**) The ESI-Q-TOF-MS-MS spectrum of the reaction product I with a retention time of 7.5 min in HPLC. (**E**) The ESI-Q-TOF-MS spectrum of reaction product II with a retention time of 8.5 min characterized with 2-chlorine isotope clusters at *m/z* 326. (**F**) The ESI-Q-TOF-MS spectrum of reaction intermediate with a retention time of 10.6 min with 2 chlorine isotope clusters at *m/z* 352.

NHPI was found to quickly disappear during its reaction with TCBQ, with the concurrent formation of a major product (here simply referred as Product I, with a retention time of 7.5 min), a minor product (Product II, with a retention time of 8.5 min), and a transient intermediate (with a retention time of 10.6 min) (Fig. 3A,B).

To better understand the underlying molecular mechanism of this reaction, the final products of the NHPI-TCBQ reaction were identified using HPLC-ESI-MS analysis. Interestingly, Product I was characterized by the same an ion peak at *m/z* 162 [(M-H)^−^] as NHPI. The molecular weight of Product I was further verified with high resolution FT-ICR-MS (*m/z* = 162.01966). MS/MS analysis revealed that NHPI and Product I have different chemical structures (Fig. 3C,D). These results suggest that Product I is an isomer of NHPI.

ESI-MS analysis revealed that Product II was characterized by an ion peak at *m/z* 326 [(M-H)^−^]. The results from both the MS (Fig. 3E) and FT-ICR-MS (SI Table S1) analysis showed that Product II contains two chlorine atoms and one nitrogen atom. However, its exact structure is not immediately clear at this initial stage (for more information on how Product II was finally identified, see below).

To determine the exact chemical structure of Product I, semi-preparative HPLC was employed to isolate and purify this compound. Although we tried our best to optimize the experimental conditions, we were unable to isolate a sufficiently pure sample of Product I because it was always accompanied by another compound, as determined by nuclear magnetic resonance (NMR) analysis (this was later found to be due to the hydrolysis of Product I during the purification process; see below for details). In spite of this interference, we deduced from the NMR results that Product I should contain a benzene ring (Ph-H, 7.26, 7.30, 7.73, 7.90 ppm) and a hydrogen atom attached to the nitrogen atom (N-H, 9.26 ppm).

Based on the results described above, we speculated that this major product should possess one of the three possible isomeric structures, as shown below:


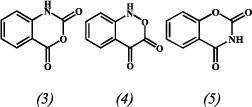


The (*3*) isomer, isatoic anhydride (IA), is a commercially available material important for organic synthesis. The (*4*) isomer is an unstable compound due to its structure. The (*5*) isomer, 2H-1,3-benzoxazine-2,4(3H)-dione, is also a commercially available material. Fortunately, through the comparison with the two authentic standards, this major product was finally identified as IA by HPLC (SI Figure S1) and ^1^H-NMR analysis (SI Figure S2). These results suggest that TCBQ can readily induce the isomerization of NHPI to IA.

### Water was involved in the isomerization of NHPI to IA induced by TCBQ: oxygen-18 isotope-labeling studies determined that water was the source and origin of oxygen for IA

Based on our previous findings and the results described above, we speculated that the anionic form of NHPI may react with TCBQ via nucleophilic substitution to initially form a transient intermediate NHPI-NO-TrCBQ:


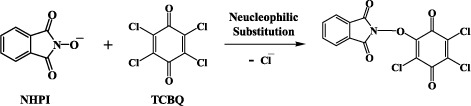


**Figure 4:**
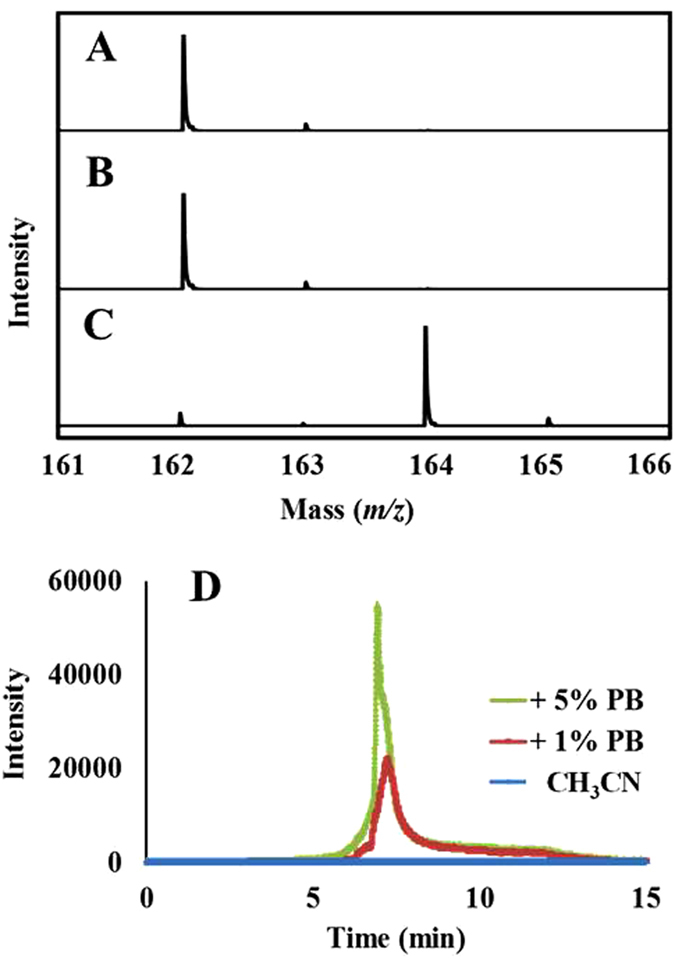
Water was involved in the isomerization of NHPI to IA induced by TCBQ: the source and origin of oxygen for IA was found to be from water. (**A**) IA generated from the reaction of NHPI and TCBQ in H_2_O. (**B**) IA isolated from the reaction of NHPI and TCBQ in H_2_O, and then dissolved in H_2_^18^O. (**C**) IA generated from the reaction of NHPI and TCBQ in H_2_^18^O. (**D**) No IA was found in the mixture of NHPI and TCBQ in pure CH_3_CN, while a detectable amount of IA was found with HPLC-TSQ-MS with the addition of 1% or 5% phosphate buffer (PB, pH = 7.4).

The oxygen atom of the N-O group should then be transferred to the quinone ring through N-O cleavage to form the initial transient product TrCBQ-OH which was already identified by HPLC/MS^17^. If this is the case, then the oxygen atom in IA should be derived from the reaction medium, where water was the most probable source. If this hypothesis is correct, then the mass spectra of the molecular ion region of IA obtained with unlabeled and oxygen-18-labeled H_2_O, should indicate a 2 mass unit-shift of the molecular ion isotope cluster peaks of the unlabeled compounds, as could be expected for the incorporation of ^18^O. Because TrCBQ-OH can also react with NHPI to generate IA, we speculated that the oxygen-18-labeled H_2_O experiment should also apply to the TrCBQ-OH/NHPI reaction. We found this to be indeed the case when oxygen-18-labeled water was used to prepare the reaction buffer for the MS analysis. To further confirm that water was essential for the TCBQ/NHPI reaction, we compared the reaction between TCBQ and NHPI in pure CH_3_CN and CH_3_CN with trace amounts of phosphate buffer. In pure CH_3_CN, NHPI and TCBQ did not form the IA reaction product; however, when 1% buffer was added, MS analysis revealed that a small amount of IA was produced. Analogous results were observed in the TrCBQ-OH/NHPI reaction (Fig. 4).

The above results clearly demonstrate that the source and origin of oxygen for IA was directly from water, and water was indeed involved in the isomerization of NHPI to IA induced by TCBQ.

### Possible pathways for the formation of IA

How is IA formed? It has been hypothesized^20^ that one of the possible pathways for the formation of IA may be through the intramolecular nucleophilic addition of *o*-carboxyl benzyl isocyanate, which can be considered as an open-loop isomer of NHPI:


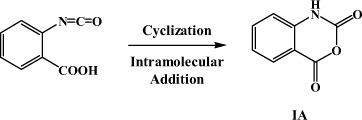


This leads to the following question, “In what way could the transient product *o*-carboxyl benzyl isocyanate be formed from NHPI?” It has been shown that one typical way could be through the Lossen rearrangement, a well-known reaction describing the conversion of an *O*-activated hydroxamic acid (R-C(O)-NH-OX) into the corresponding isocyanate^21^,^22^. The rate-limiting step of this reaction is the activation of the hydroxamic acid by a variety of activating agents (i.e., sulfonyl and benzoyl chloride, etc.; X = SO_2_R, C(O)R):


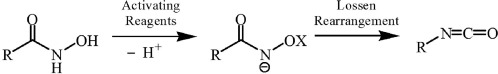


The loss of a proton from the nitrogen atom to form the anionic intermediate under alkaline conditions is considered to be essential for the classic Lossen rearrangement. Indeed, we recently found that benzohydroxamic acid can be activated by halogenated quinones (XBQs) to produce phenyl isocyanate, which requires the formation of an anionic N intermediate^17^,^23^. However, in the present study, the anionic N intermediate cannot be formed by losing a proton because the N atom is linked to two carbonyl groups. It has been reported that *O*-activated *N*-hydroxyphthalimide could also undergo Lossen rearrangement^24^,^25^,^26^, but various bases were required to trigger the ring-opening of the adduct that typically occurs in organic solutions. Evidence from the analysis described above and the results from oxygen-18 isotope-labeling for direct water involvement suggest that the reaction between NHPI and TCBQ may proceed through a previously unknown Lossen-type rearrangement pathway.

### The facile two-step isomerization of NHPI to IA can also be induced by other halogenated quinoid compounds

**Figure 5:**
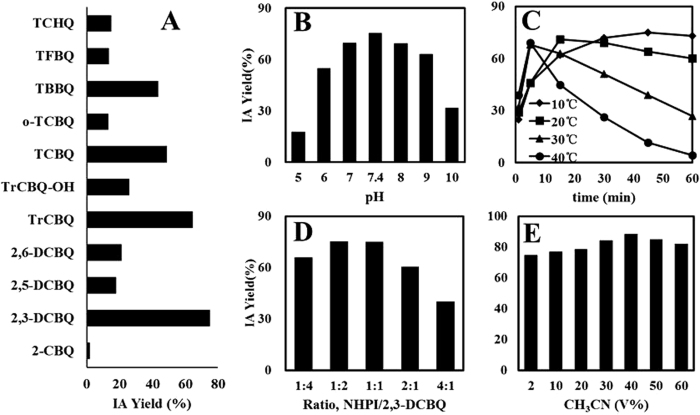
The facile isomerization of NHPI to IA can also be induced by other halogenated quinoid compounds. (**A**) IA could be generated from the reaction of NHPI and other halogenated quinones. (**B–E**) The optimization of experimental conditions for maximum IA production from the reaction of NHPI and 2,3-DCBQ: pH, 7.4; CH_3_CN, 40% (V%); T, 10 °C; NHPI: 2,3-DCBQ. (**B**) NHPI, 0.2 mM; 2,3-DCBQ, 0.2 mM; 5 min; 30 °C; (**C**) NHPI, 0.2 mM; 2,3-DCBQ, 0.2 mM; pH, 7.4; (**D**) NHPI, 0.2 mM; 2,3-DCBQ, 0.8, 0.4, 0.2, 0.1, and 0.05 mM; pH, 7.4; 45 min; and (**E**) NHPI, 0.2 mM; 2,3-DCBQ, 0.4 mM; pH, 7.4; 10 °C; 45 min.

We found that the facile isomerization of NHPI to IA can also be induced when TCBQ was substituted with other tetrahalogenated quinoid compounds, such as tetrafluoro-1,4-benzoquinone (TFBQ), tetrabromo-1,4-benzoquinone (TBBQ), tetrachloro-1,2-benzoquinone (*o*-TCBQ), and tetrachloro-1,4-hydroquinone (TCHQ). IA can also be produced from NHPI by less chlorinated quinones, such as trichloro-1,4-benzoquinone (TrCBQ), trichloro-2-hydroxy-1,4-benzoquinone (TrCBQ-OH), 2,3-dichloro-1,4-benzoquinone (2,3-DCBQ), 2,5-dichloro-1,4-benzoquinone (2,5-DCBQ), 2,6-dichloro-1,4-benzoquinone (2,6-DCBQ) and 2-chloro-1,4-benzoquinone (2-CBQ) (Fig. 5A; SI Figure S3). Using the HPLC-ESI-TSQ-MS method coupled with selected reaction monitoring (SRM) mode and the commercially available pure IA as standard, we found that among all the quinoid compounds tested, 2,3-DCBQ gives the highest IA yield (88%) (Fig. 5B–E).

### Product II was identified as the reaction product between two transient reaction intermediates, TrCBQ-OH and anthranilic acid

While studying the time course of IA generation, we noticed that IA was produced quickly and then slowly degraded (Fig. 5C). Because it has been shown that IA hydrolyzes into anthranilic acid in dilute alkaline solutions^27^, we speculated that a similar hydrolysis may occur under our conditions. HPLC-ESI-MS analysis using authentic anthranilic acid as the standard confirmed that this was indeed the case (SI Figure S4).

As shown above, TCBQ was first hydrolyzed to TrCBQ-OH and then to DDBQ. The yield of DDBQ was quite different when using either TCBQ (65%) or TrCBQ-OH (nearly 100%) (Fig. 2A,B) as the starting chemical, indicating that a side reaction may occur in the NHPI/TCBQ system. After carefully screening all possible species involved in this reaction, anthranilic acid was considered to be the most likely to react with TrCBQ-OH to produce the mysterious Product II via nucleophilic substitution^28^,^29^. Fortunately, we found that this is true. Using MS, we determined that Product II was 2,5-dichloro-3-(*N*-2-carboxyl phenyl)-6-hydroxy-1,4-benzoquinone (SI Figure S5).


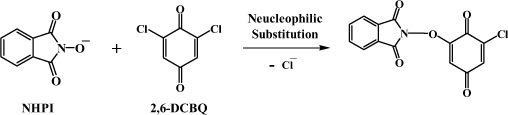


### The molecular mechanism of the two-step isomerization of NHPI to IA induced by TCBQ: a water-assisted double Lossen rearrangement coupled with rapid intramolecular nucleophilic addition and cyclization

**Figure 6:**
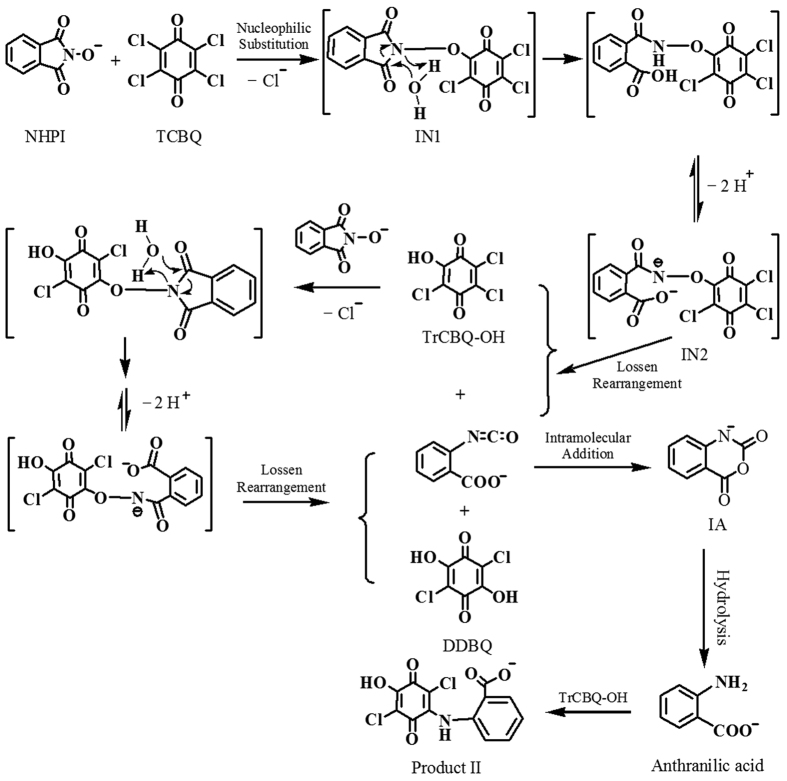
Proposed molecular mechanism for the two-step isomerization of NHPI to IA induced by TCBQ: a water-assisted double Lossen rearrangement coupled with rapid intramolecular nucleophilic addition and cyclization.

Based on the findings of the current study, earlier research on Lossen rearrangement^17^,^21^,^22^,^23^, the fact that *N*-hydroxy phthalimide anion is a particularly effective nucleophile, and the involvement of water, we proposed a unique TCBQ-activated and water-assisted Lossen rearrangement mechanism for the isomerization of NHPI to IA (Fig. 6).

According to this mechanism, a nucleophilic reaction took place between the *N*-hydroxy phthalimide anion (NHPI-NO^−^) and TCBQ, forming an unstable transient intermediate (IN1) NHPI-NO-trichloro-1,4-benzoquinone. Following the attack of water on intermediate IN1, the anionic intermediate IN2 was formed via the loss of two protons from the nitrogen atom and the carboxylic group; then, a spontaneous Lossen rearrangement led to the formation of TrCBQ-OH and *o*-carboxyl benzyl isocyanate, which was a short-lived open-loop isomer of NHPI. IA, the re-loop-locked isomer of NHPI, is then quickly formed via rapid intramolecular nucleophilic addition and cyclization. When NHPI is in excess, TrCBQ-OH further reacts with NHPI through a similar reaction intermediate and a second-step water-assisted Lossen rearrangement reaction, yielding DDBQ and another molecule of IA. The minor Product II was produced via the nucleophilic substitution between TrCBQ-OH and anthranilic acid.

**Figure 7:**
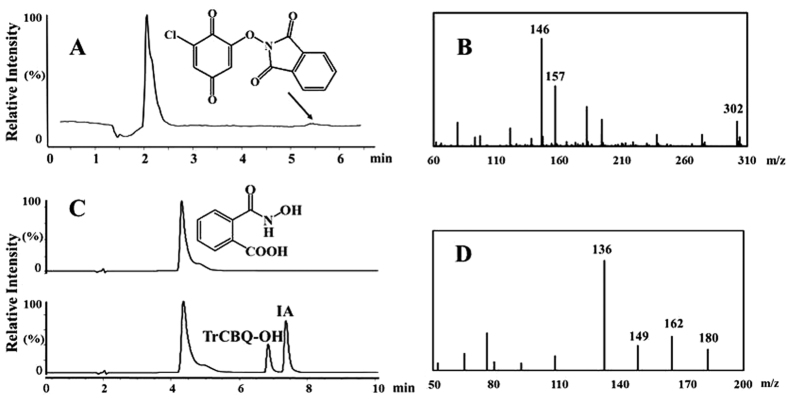
Identification of the NHPI/2,6-DCBQ adduct and the formation of IA from the interaction between *o*-carboxy benzohydroxamic acid and TCBQ. (**A**) TIC chromatography of the reaction between NHPI and 2,6-DCBQ; the t_R(adduct)_ was 5.5 min. (**B**) MS/MS spectrum of the NHPI/2,6-DCBQ adduct (*m/z*, 302); (**C**) TIC chromatography of *o*-carboxy benzohydroxamic acid and its reaction with TCBQ to produce IA; and (**D**) MS/MS spectrum of the *o*-carboxy benzohydroxamic acid (*m/z*, 180).

It should be noted that the postulated reaction intermediate NHPI-NO-trichloro-1,4-benzoquinone (IN1) and the rearranged initial product *o*-carboxy phenyl isocyanate could not be isolated and identified directly, possibly due to their extreme instability and reactivity. It has been shown that the rate-limiting step in the Lossen rearrangement is the activation of the hydroxamic acid and that the rate of the rearrangement is directly proportional to the relative acidity of the conjugate acid of the anionic leaving group^21^,^22^. Due to the strong acidity of TrCBQ-OH (pK_a_: 1.10) and DDBQ (pK_a1_: 0.58; pK_a2_: 2.72)^18^,^30^,^31^,^32^, the conjugate acids of the anionic leaving groups in the present study, it is expected that the rate of rearrangement of the postulated reaction intermediate (IN1) should be very fast. However, when TCBQ was substituted with 2,6-DCBQ, the acidity of the conjugate acid of the anionic leaving group, 2-chloro-6-hydroxy-benzoquinone, was much weaker (pK_a_ = 3.65)^23^. We would then expect that the 1:1 substitution adduct of NHPI/2,6-DCBQ should be stable enough for isolation and identification by HPLC-MS. We found that this was indeed the case (Fig. 7A,B).


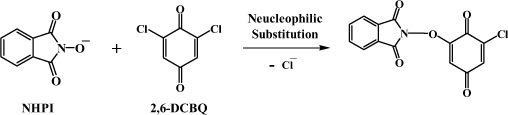


Based on our previous work^17^,^23^, we expected that *o*-carboxy benzohydroxamic acid could be readily activated by TCBQ to generate *o*-carboxy phenyl isocyanate via Lossen rearrangement. If the mechanism proposed above for the formation of IA via the *o*-carboxy phenyl isocyanate intermediate were correct, then IA should also be produced from *o*-carboxy benzohydroxamic acid activated by TCBQ. We found that this is true (Fig. 7C,D). These results strongly support that *o*-carboxy phenyl isocyanate is the intermediate in the formation of IA from the reaction between NHPI and TCBQ.


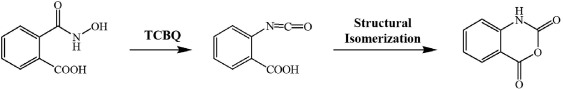


### Why this isomerization and rearrangement reaction is so unusual?

Isomerization, which plays an important role in the development of chemistry, material science, pharmacology, biology and medicine^30^,^31^,^32^, can be divided into three major types: constitutional, configurational and conformational^33^. The transformation of NHPI to IA should be of the constitutional isomerization form, also known as structural isomerization.

Characteristically, isomerizations are rearrangements that leave the carbon skeleton intact but change the positions of the substituents or functional groups in space. In an isomerization, the molecular formulas of the reactant and product are always the same; in a rearrangement, they can be either the same (as in the case for Beckmann rearrangement), or different (as for Lossen rearrangement)^22^. The Lossen rearrangement is typically carried out using a hydroxamic acid as the starting chemical and a corresponding isocyanate as the rearrangement product, which are 18 units (H_2_O) lower than the molecular weight of the hydroxamic acid. However, the present findings showed that the Lossen rearrangement product IA is also an isomer of the starting chemical NHPI.

The most surprising finding in this study was that TCBQ and other halogenated quinones (XBQs) could activate NHPI to go through a water-assisted Lossen rearrangement coupled with intramolecular cyclization, leading to the formation of its isomer, IA. Compared to the classic Lossen rearrangement, this newly discovered rearrangement and isomerization has the following unique characteristics: (i) In a classic Lossen rearrangement, a base is required to transform the *O*-activated hydroxamic acid into the critical anion nitrogen intermediate, the essential driving force for the rearrangement. However, in this NHPI/XBQs system, bases were not required for transforming the adduct into its corresponding nitrogen anion. Because the strong electron-withdrawing trichloroquinoid group significantly increased the electrophilicity of the carbonyl group for the transient intermediate NHPI-NO-trichloro-1,4-benzoquinone (IN1)^34^, this made the carbonyl group much more prone to attack by water, the weak nucleophile in the solvent. In other words, the essential nitrogen anion was produced only through the assistance of water. (ii) Interestingly, we found that the rearranged isocyanate transient product of NHPI/XBQs turned out to be an open-loop isomer of NHPI as a result of the participation of water. This short-lived isocyanate intermediate was then quickly converted into its corresponding loop-locked isomer IA via intramolecular addition, making this water-assisted Lossen rearrangement also a very unique isomerization. In contrast, the classic Lossen rearrangement involves a typical dehydration process. (iii) Most of the previously reported Lossen rearrangement reactions occur only under alkaline conditions and/or through heating to a requisite temperature^21^. In the present study, we found that the reaction between NHPI and TCBQ could occur at a normal physiological temperature and under neutral or even a weakly acidic pH, therefore making these new findings more biologically and environmentally relevant. To our knowledge, this is the first report demonstrating that NHPI could be isomerized to IA via XBQ-mediated and a previously unknown water-assisted Lossen rearrangement coupled with rapid intramolecular addition and cyclization under physiological conditions.

### A novel method for the preparation of IA from NHPI

IA, known for over a century, is an extremely versatile compound that easily reacts with both electrophiles and nucleophiles, lending itself to a wide range of applications in the manufacturing of agricultural chemicals, dyes, fragrances, pharmaceuticals and miscellaneous industrial chemicals^28^. Recently, IA derivatives, mainly *N*-methyl-IA and 1-methyl-7-nitro-IA, were also used to alter the ribose moiety of tRNA and mRNA for further structural and functional studies^35^,^36^. Three types of reactions have been commonly used to prepare IA: (1) cyclization of anthranilic acid with carbonic acid derivatives, (2) oxidation of isatin in glacial acetic acid and (3) rearrangement of phthalic acid derivatives (SI Scheme S1)^37^.

Some of these methods have been successfully applied in industrial production. However, they usually work under alkaline or acidic conditions and/or through heating. Furthermore, hypertoxic chemicals such as phosgene, chromium trioxide and chloroformate were also involved in these methods. Here, we developed a new method for the synthesis of IA from NHPI. Compared to traditional methods, this reaction could occur in water at room temperature and under neutral or even weakly acidic pH. The activating reagent TCBQ (also called *p*-chloranil) is readily available commercially, and its main final product is the non-toxic dihydroxylation product DDBQ. These features make this method more environmentally friendly.

We furthered our investigation by synthesizing other derivatives of IA using this method. Substituted *N*-hydroxyphthalimides were synthesized from their corresponding phthalic anhydrides with a simplified microwave-assisted synthetic method^38^. Appreciable amounts of the corresponding substituted IAs were easily produced after reaction with 2,3-DCBQ in phosphate buffer (pH = 7.4), which are listed below:


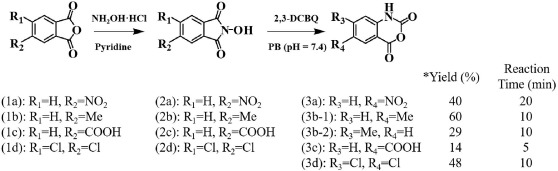


To our knowledge, this is also the first report showing that TCBQ and other halogenated quinones can serve as a unique class of activating agents for the activation of NHPIs to produce its isomer IAs under very mild experimental conditions.

### Potential biological and environmental implications

We found that this unusual isomerization of NHPI coupled with concurrent detoxication of TCBQ to its much less toxic hydroxylation product is not only limited to TCBQ and NHPI; it is also a general mechanism for all halogenated quinoid compounds. Therefore, our findings may have interesting biological and environmental implications. Many widely used halogenated aromatic compounds (which are considered probable human carcinogens) including halogenated phenols, Agent Orange and hexachlorobenzene, can be metabolized *in vivo*^2^,^5^,^39^, or dehalogenated enzymatically and chemically^7^,^8^, to their corresponding quinones. Chlorinated quinoid compounds were also detected in pulp and paper mill discharge^5^. More recently, more than a dozen of halogenated quinones (suspected to be bladder carcinogens) were characterized as chlorination disinfection byproducts in drinking and in swimming pool water^6^. These halogenated quinones not only induce oxidative damage in DNA and other macromolecules but also form protein and DNA conjugates both *in vitro* and *in vivo*^1^,^2^,^5^. Thus, these molecules are potential mammalian carcinogens, which render their remediation or destruction under mild conditions of critical importance.

In our previous studies, we found that TCBQ and H_2_O_2_ can produce highly reactive hydroxyl radicals via a metal-independent mechanism^5^,^6^,^40^,^41^,^42^,^43^, which can cause oxidative damage to DNA and other macromolecules^44^,^45^,^46^,^47^. Based on our finding that NHPI can effectively detoxify TCBQ, we expect that NHPI should also effectively protect plasmid pBR322 DNA from single-strand and double-strand breakage induced by TCBQ/H_2_O_2_. We found that this is indeed the case (SI Figure S6). Our present and previous studies demonstrated that^11^,^17^,^48^,^49^ NHPI and other hydroxamic acids may also be especially suited for detoxifying halogenated quinones. Further research is needed to investigate whether NHPI and other hydroxamic acids could be used safely and effectively as prophylactics for the prevention or treatment of human diseases, such as liver and bladder cancer associated with carcinogenic halogenated quinoid compounds.

In summary, the molecular mechanism of the detoxication of TCBQ by the well-known organocatalyst NHPI was elaborated in detail using diverse HPLC-MS and NMR spectroscopic methods. In particular, the oxygen-18 isotope labeling experiment and the addition of a mild buffer into the CH_3_CN solvent played a critical role in determining the participation of water. The data showed that with the assistance of water, TCBQ effectively induced NHPI into its open-loop isomer *o*-carboxyl benzyl isocyanate through an unusual Lossen rearrangement and subsequently into its loop-locked isomer IA quickly through an intramolecular addition. This finding enriched our knowledge of the rearrangement discovered over a century ago. Along with the structural isomerization of NHPI, TCBQ was dechlorinated and detoxicated when NHPI was in excess. Surprisingly, the IA yield under optimized conditions (88%) was as high as those achieved through traditional synthetic methods. These findings provide a valuable foundation for further studies in related fields.

## Methods

### Chemicals

Tetrachloro-1,4-benzoquinone (TCBQ; tetrachloro-*p*-benzoquinone or *p*-chloranil), *N*-hydroxyphthalimide (NHPI), and all other chemicals were purchased from Sigma. The oxygen-18-enriched H_2_O (90%, [^18^O]-H_2_O) was purchased from J&K. The chemicals were used as received without further purification. All stock solutions (20 mM) were prepared by dissolving the chemicals in CH_3_CN.

### Electronic Spin Resonance (ESR) Studies

The basic system used in this study consisted of TCBQ and NHPI dissovled in CH_3_CN, and the spin-trapping agent DMPO (100 mM). Reactions were done in 100 mM Chelex-treated phosphate buffer (pH 7.4) at 37 °C. All reaction mixtures were air-saturated for the ESR experiments. ESR spectra were recored 1 min after the interactions between TCBQ and NHPI on a Bruker (Billerica, MA) ER 200 D-SRC spectrometer operating at 9.8 GHz and a cavity equipped with a Bruker Aquax liquid sample cell. Typical spectrometer parameters were scan range, 100G; field set, 3509G; time constant, 200 ms; scan time, 60 s; modulation amplitude, 1.0G; modulation frequency, 100 kHz; receiver gain, 1.25 × 10^4^; and microwave power, 9.8 mW.

### Identification of the Reaction Products of TCBQ

The reactions were carried out by mixing the corresponding chemicals in Chelex-treated phosphate buffer (100 mM, pH 7.4). The reaction products of TCBQ/NHPI were analyzed using high-performance liquid chromatography combined with electrospray ionization quadrupole time-of-flight mass spectrometry (HPLC-ESI-Q-TOF-MS, Agilent 6540, USA). The HPLC system was equipped with a photodiode array detector. FT-ICR-MS was used to accurately identify the molecular weight of the reaction products. For MS analysis, 100 μL of 0.5 mM TCBQ reaction solution with 1 mM NHPI in Chelex-treated CH_3_COONH_4_ buffer (100 mM, pH 7.4) at 37 °C during the 0–30 min reaction period was injected into an LC-18 C_18_ column (5 μm, 4.6 × 250 mm) eluted with the mobile phase at a rate of 1.0 mL/min. The chromatographic eluent was then led to the mass spectrometer through a splitter. The DDBQ yield from TCBQ/NHPI was quantified with HPLC using commercial-grade DDBQ as the standard. The yields of TrCBQ-OH were quantified using HPLC-ESI-TSQ-MS in SRM mode due to its poor degree of separation in HPLC.

### Isolation and Identification of NHPI Reaction Products

#### Isatoic Anhydride (IA)

0.2 mM TCBQ and 0.4 mM NHPI were mixed in PB (pH = 7.4). No precipitate was observed during the reaction. The ethyl acetate extraction solution of the reaction mixture could not be effectively separated with analytical thin-layer chromatography (TLC) plates either. Milligram scale collection of Product I was performed using a semi-preparative HPLC apparatus equipped with a UV detector. The reaction solution of TCBQ/NHPI (1:2, 1 mM TCBQ was added slowly drop-wise) in 8 mL of Chelex-treated phosphate buffer (100 mM, pH 7.4) and 2 mL of CH_3_CN at 37 °C after a reaction time of 10 min was filtered and then injected into a Prep-C18 semi-preparative HPLC column (15 cm × 10.0 mm, 3 μm). The conditions were as follows: mobile phase: A, 1% formic acid, pH 2.1; B, CH_3_CN; flow rate: 20 mL/min; column temperature, 25 °C; and gradient elution (the gradient used was a linear gradient of 10% B held from 0 to 2 min, 10–30% B from 2 to 5 min, 30% B held from 5 to 10 min, 30–10% B from 10 to 13 min, and 10% B maintained from 13 min to 15 min for the column to re-equilibrate before the subsequent injection). The fractions were monitored at 316 nm and collected manually. The collected fractions were then evaporated to eliminate CH_3_CN and then extracted with ethyl acetate. The collected ethyl acetate layer was evaporated under a vacuum until dry. The solid was then further freeze-dried with a lyophilization. The ^1^H NMR spectrum of Product I was acquired using a Bruker DPX-400 spectrometer at 400 MHz with tetramethylsilane ((CH_3_)_4_Si) as the internal standard and CH_3_CN-*d3* as the solvent. Although the purity was not adequate, we recognized that Product I should contain a benzene ring (Ph-H, 7.26, 7.30, 7.73, 7.90 ppm) and a reactive hydrogen atom (N-H, 9.26 ppm). Product I was confirmed to be IA by comparing the NMR spectrum to that of the commercially available IA (Ph-H, 7.25, 7.29, 7.71, 7.98 ppm, N-H, 9.24 ppm). When we further applied this reaction to other halogenated quinones, 2,3-DCBQ was found to give the highest IA yield (88%). The yield was quantified using the HPLC-ESI-TSQ-MS method with authentic IA as the standard in SRM mode (the ion pair selected was *m/z* 162–118) due to its poor degree of separation in HPLC. The optimal condition was: pH, 7.4; CH_3_CN, 40% (V%); T = 10 °C; NHPI: 2,3-DCBQ, 1:1~1:2; t = 45 min.

### Oxygen-18 Isotope-labeling Experiment

To investigate the source and origin of the oxygen atom inserted into IA formed from the reaction between NHPI and TCBQ (or TrCBQ-OH), 0.5 mM TCBQ (or TrCBQ-OH) was incubated with NHPI in oxygen-18-enriched H_2_O ([^18^O]-H_2_O) in a final volume of 0.1 mL of CH_3_COONH_4_ buffer (0.1 M, pH 7.4). A control experiment with unlabeled H_2_O was also performed. After 1.0 min of incubation, the samples were analyzed using HPLC-ESI-Q-TOF-MS as described above.

### Synthesis of *o*-carboxy Benzohydroxamic Acid

2 mM of NHPI was dissolved in 50 mL pure water. The solution was continuously stirred using a magnetic stirrer. A slight excess of NaOH solution was added to the solution drop-wise. The NHPI solution turned yellow and then became colorless after several minutes of hydrolysis. The solution was quickly acidified with a high concentration of HCl and then dried with a freeze drier. Powdered white solid was obtained. The crude *o*-carboxy benzohydroxamic acid was identified using the HPLC-MS method.

### Synthesis of IA Derivatives

A mixture of substituted phthalic anhydrides (2 mmol), hydroxylamine hydrochloride (4 mmol) and pyridine (20 mmol) in a 200 mL round-bottom flask was irradiated in a domestic microwave oven (2450 MHz, 700 W) for 20 s. Pyridine was removed under reduced pressure. The residue was cooled to 0 °C, and then 1 M HCl (10 cm^3^) was added. The yellow precipitate was filtered, washed with water (10 cm^3^), and dried *in vacuo* to yield the corresponding NHPI derivatives. The NHPI derivatives obtained were used to react with 2,3-DCBQ in phosphate buffer (pH = 7.4) at room temperature. The IA derivatives generated were identified using the HPLC-MS method and then roughly quantified with HPLC. The mobile phase was 30% A (0.1% formic acid, aq) and 70% B (acetonitrile). The *m/z* and λ_max_ of the IAs were as follows: 3a (*m/z* = 207, λ_max_ = 310 nm, t_R_ = 14.5 min); 3b-1 (*m/z* = 176, λ_max_ = 326 nm, t_R_ = 7.2 min); 3b-2 (*m/z* = 176, λ_max_ = 314 nm, t_R_ = 6.6 min); 3c (*m/z* = 206, λ_max_ = 336 nm, t_R_ = 3.1 min); and 3d (*m/z* = 230, λ_max_ = 330 nm, t_R_ = 8.6 min).

### DNA Damage

The conversion of the covalently closed circular double-stranded supercoiled DNA to a relaxed open circle form and a linear form was used to investigate DNA strand breakage induced by TCBQ and H_2_O_2_. The experiment was conducted by incubating the plasmid pBR322 DNA (0.5 μg/mL) at 37 °C for 1 h in Chelex-treated sodium phosphate buffer (100 mM, pH 7.4) with the TCBQ/H_2_O_2_ system in the absence or presence of the indicated concentrations of NHPI.

## Additional Information

**How to cite this article:** Li, F. *et al*. An Exceptionally-Facile Two-Step Structural Isomerization and Detoxication via a Water-Assisted Double Lossen Rearrangement. *Sci. Rep.* **6**, 39207; doi: 10.1038/srep39207 (2016).

**Publisher's note:** Springer Nature remains neutral with regard to jurisdictional claims in published maps and institutional affiliations.

## Supplementary Material

Supplementary Information
