# Supplementary material for: An Exceptionally Facile Two-Step Structural Isomerization and Detoxication via a Water-Assisted Double Lossen Rearrangement
Source: Sci Rep. 2016 Dec 23;6:39207. doi: 10.1038/srep39207 (PMC5180244; doi:10.1038/srep39207)
Supplement: Supplementary Information [file srep39207-s1.pdf]

Supporting Information

An Exceptionally-Facile Two-Step Structural Isomerization and Detoxication via a Water-Assisted Double Lossen Rearrangement

Feng Li[1], Chun-Hua Huang[1], Lin-Na Xie[1], Na Qu[1], Jie Shao[1], Bo Shao[1] & Ben-Zhan Zhu[*,1,2]

[1.] State Key Laboratory of Environmental Chemistry and Eco-toxicology, Research Centre for Eco-environmental Sciences, the Chinese Academy of Sciences, Beijing 100085, PR China.

[2.] Linus Pauling Institute, Oregon State University, Corvallis, OR 97331, USA

Correspondence and requests for materials should be addressed to B. Z. Z. (email:bzhu@rcees.ac.cn)

*Corresponding author, E-mail: bzhu@rcees.ac.cn

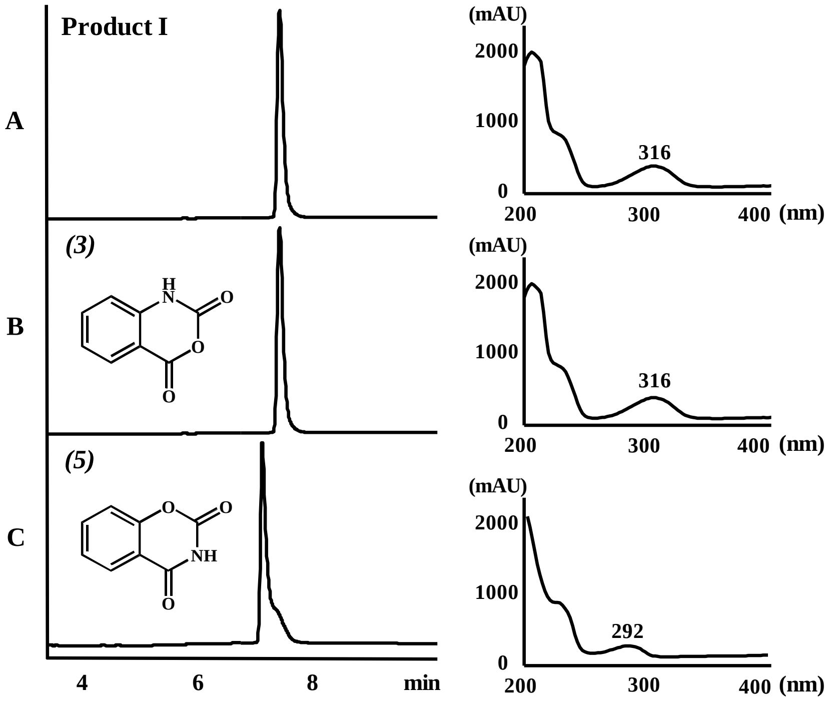

Figure S1. Product I and *(3)* have the same retention time and UV spectrum, while the retention time and UV spectrum between Product I and *(5)* are different. These HPLC data show that Product I is *(3)*. (A) Product I. (B) *(3)* (Isatoic anhydride). (C) *(5)*.

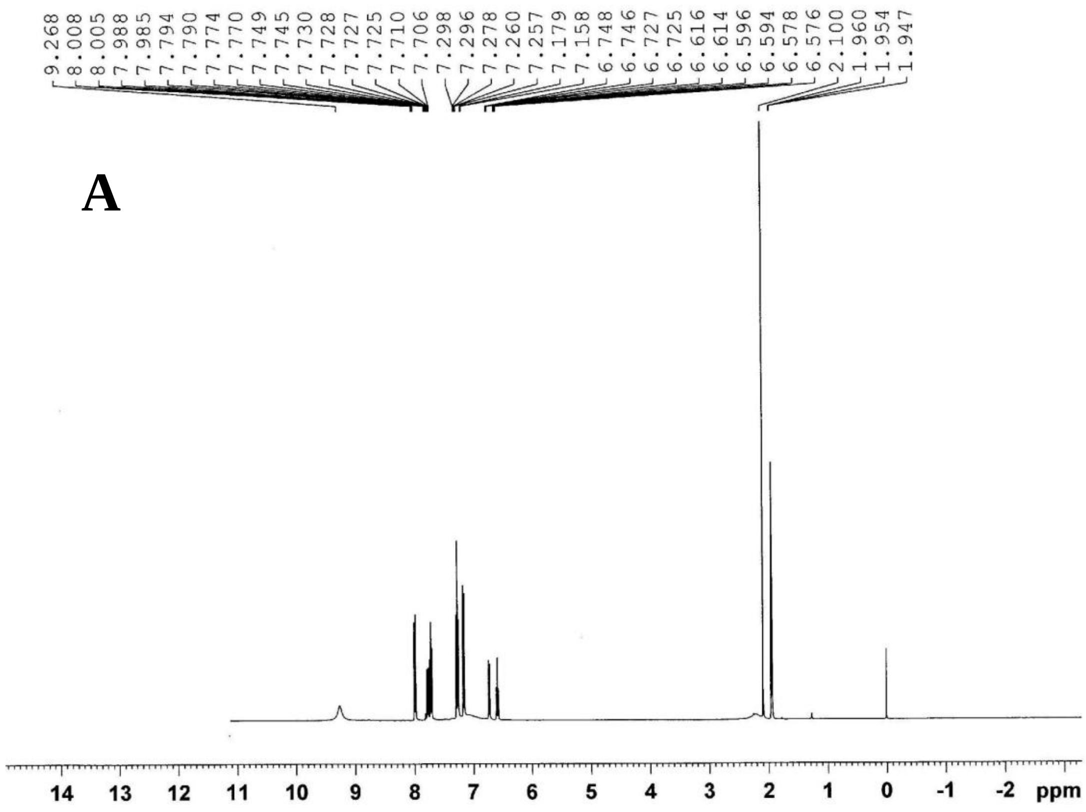

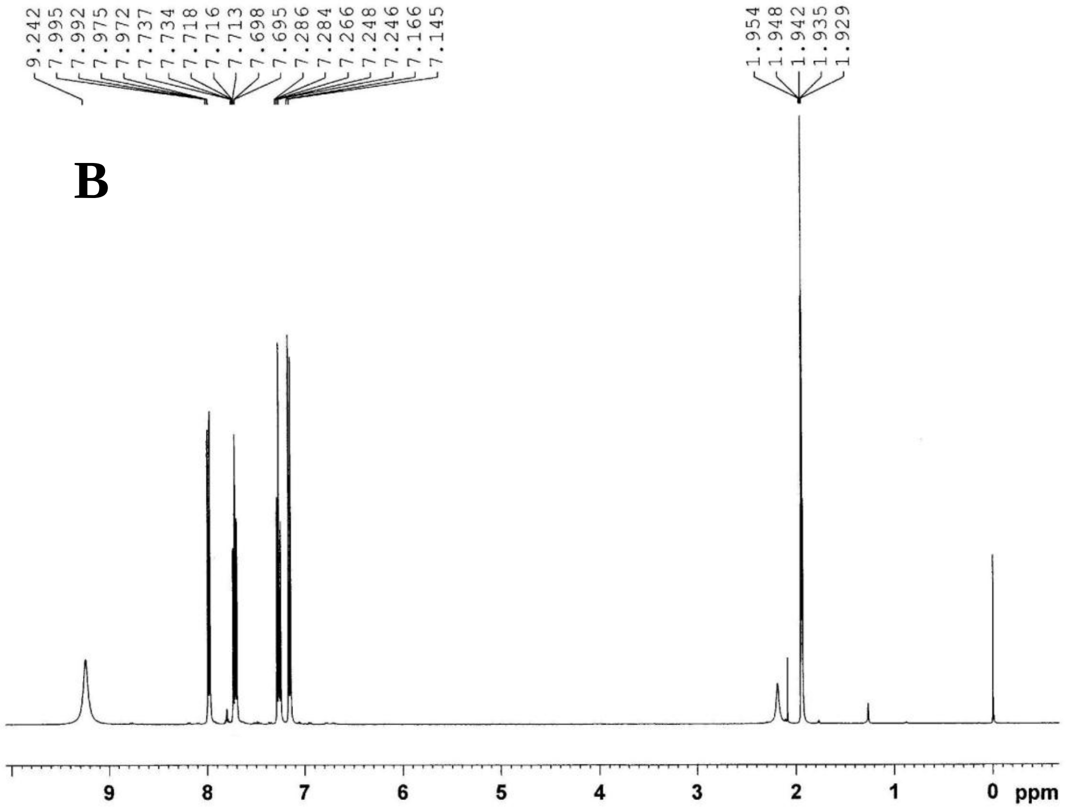

Figure S2. NMR spectra of (A) isolated Product I; (B) authentic isatoic anhydride.

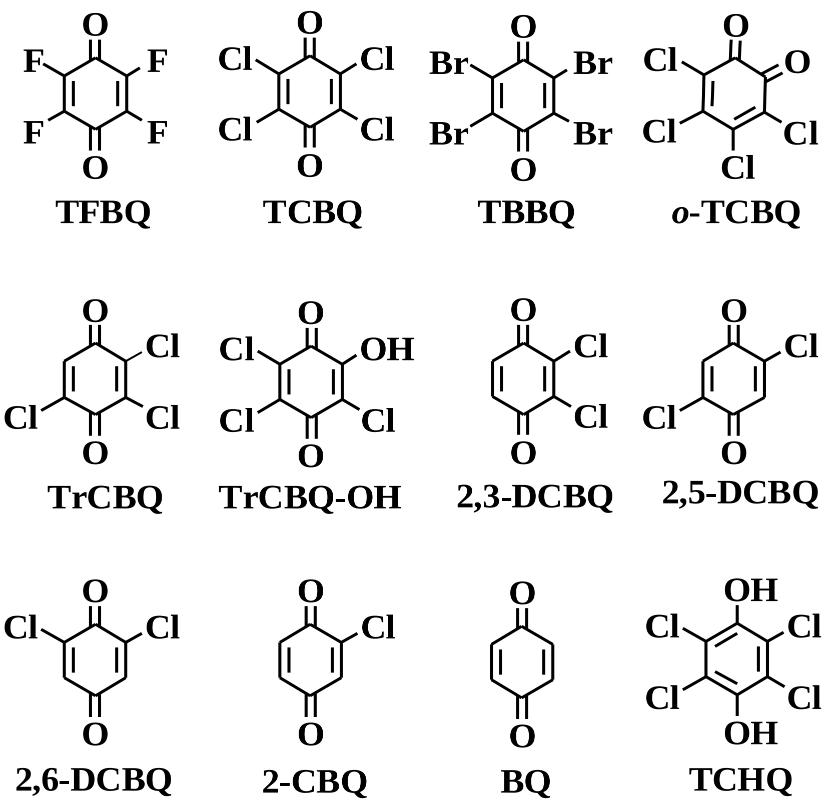

Figure S3. Chemical structures of halogenated quinoid compounds used in this study.

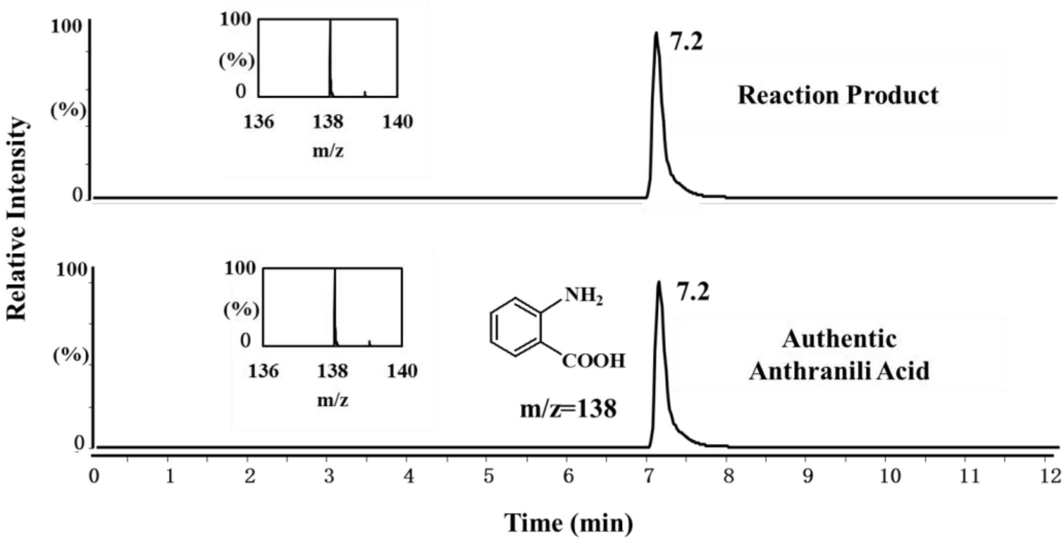

Figure S4. The hydrolysis product of IA is confirmed to be anthranilic acid (AA) by comparison with the authentic standard. The hydrolysis product of IA shows the same retention time and *m/z* in the HPLC-MS data.

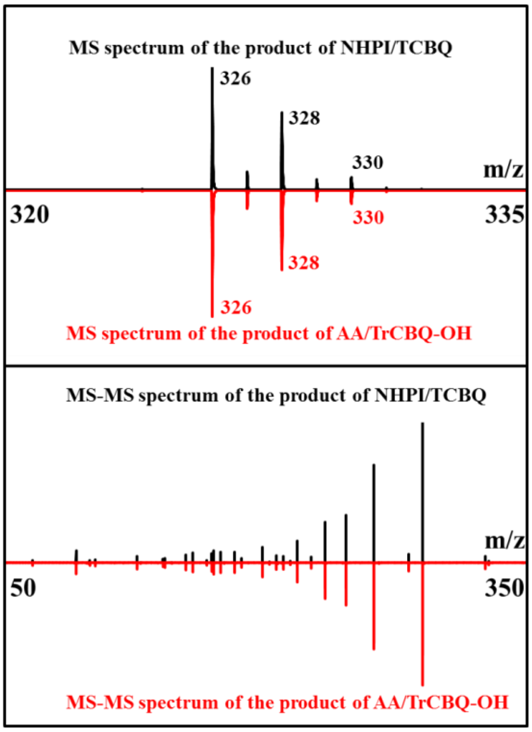

Figure S5. The MS and MSMS spectra of Product II. The MS and MSMS data of Product II of NHPI/TCBQ match well with those of the reaction of AA/TrCBQ-OH, which means that Product II is as the same as the nucleophilic product of AA/TrCBQ-OH.

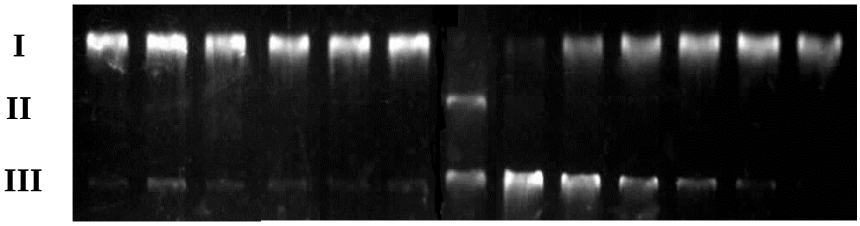

**pBR322 DNA**	**+**	**+**	**+**	**+**	**+**	**+**	**+**	**+**	**+**	**+**	**+**	**+**	**+**
**TCBQ**	**-**	**+**	**-**	**-**	**-**	**+**	**-**	**+**	**+**	**+**	**+**	**+**	**+**
**H_2O_2**	**-**	**-**	**-**	**+**	**-**	**+**	**+**	**+**	**+**	**+**	**+**	**+**	**+**
**NHPI (mM)**	**-**	**-**	**10**	**-**	**10**	**10**	**-**	**0.1**	**0.4**	**1**	**2**	**5**	**10**

Figure S6. Protection by NHPI against TCBQ/H_2O_2-induced DNA damage. The concentrations: pBR322 DNA, 5μg/mL; TCBQ, 0.1 mM; H_2O_2, 10 mM.

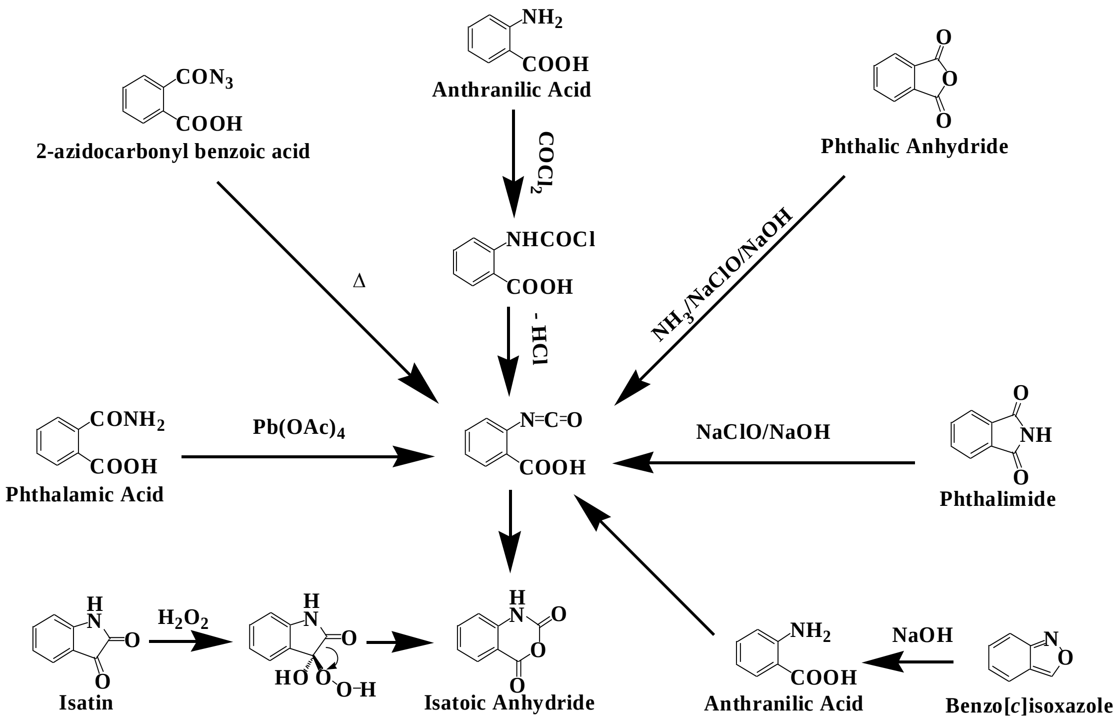

Scheme S1. The most commonly-used methods for the synthesis of isatoic anhydride.

Table S1. The FT-ICR-MS data of the products of the reaction of NHPI and TCBQ.

Meas. m/z	#	Formula	Score	m/z	Err [mDa]	Err [ppm]	mSigma	rdb	E?conf	N-rule
162.01966	1	C8H4NO3	100.0	162.01967	0.00	0.02	9.1	7.5	Even	Ok
325.96277	2	C13H6Cl2NO5	100.0	325.96285	0.08	0.26	28.3	10.5	Even	Ok
351.94198	3	C14H4Cl2NO6	100.0	351.94212	0.14	0.39	90.1	12.5	Even	Ok
